# Communication of advance care planning decisions: a retrospective cohort study of documents in general practice

**DOI:** 10.1186/s12904-020-00613-1

**Published:** 2020-07-14

**Authors:** Laura Panozzo, Pam Harvey, Meagan-Jane Adams, Dennis O’Connor, Bernadette Ward

**Affiliations:** 1 School of Rural Health, Monash University, 26 Mercy Street, Bendigo, Victoria 3550 Australia; 2 La Trobe Rural Health School, Edwards Road, Bendigo, Victoria 3550 Australia; 3 Bendigo Health, 100 Barnard Street, Bendigo, Victoria 3550 Australia

**Keywords:** Advance care planning, End-of-life care., Palliative medicine., General practice.

## Abstract

**Background:**

Doctors, particularly general practitioners, play a significant role in assisting patients to create advance care plans. When medically indicated, these documents are important tools to promote congruence between end-of-life care and patient’s personal preferences. Despite this, little is known regarding the availability of these documents in hospitals. The aim of this study was to identify the proportion of people who died in hospital *without* an advance care plan and how many of these had advance care planning (ACP) documents in their general practice records.

**Methods:**

A retrospective cohort study was conducted of patient hospital records with manual linkage to general practice records. The large regional hospital in Victoria, Australia has a catchment population in excess of 300,000 people. The study sample was patients aged 75 years and over who died in the hospital between 1 January 2016 and 31 December 2017. The hospital records of these patients were examined to identify those which did not have a system alert for ACP documents on the file. Alerted ACP documents were limited to those legislated in the state of Victoria: advance care plan, Enduring Power of Attorney (Medical Treatment) or Enduring Power of Guardianship. Where no ACP document system alert was found in the hospital record, the patient’s nominated general practice was consented to participate and the corresponding general practice record was examined. Data were analysed using descriptive statistics.

**Results:**

Of the 406 patients who died in hospital, 76.1% (309) did not have a system alert for any ACP document. Of the 309 hospital records without a system alert, 144 (46.7%) corresponding general practice records were examined. Of these, 14.6% included at least one ACP document, including four advance care plans, that were not available in hospital.

**Conclusions:**

Unless ACP documents are consistently communicated from general practice, patient’s preferences may be unknown during end-of-life care. It is important that both doctors and patients are supported to use connected electronic health records to ensure that documents are readily available to healthcare staff when they are required.

## Background

Education campaigns have aimed to increase both the awareness of advance care planning (ACP) discussions, and prevalence of ACP documents [1, 2]. Health practitioners have become more familiar and confident in their ability, to use these documents, to make decisions during end-of-life care that support patients’ preferences [3]. These documents play a particularly important role when a patient has lost decision making capacity and can no longer communicate for themselves [4, 5]. Ensuring a patient’s preferences are fulfilled is the most common priority for health practitioners during end-of-life care [3]. Engagement with, and completion of, ACP documents is influenced by cultural factors [6]. In Australia, ACP conversations can be emotionally challenging for both patients and practitioners but are of great value [7].

In Australia, ACP documents primarily include an advance care plan as a written statement of preferences or a document formally appointing a substitute decision maker; however, the specific terminology varies across jurisdictions [4, 8]. The state government of Victoria provides an ACP document template, however advance care plans can be constructed on any written form if it fulfils the relevant legal requirements [3, 5, 8]. Historically, legal representatives have also been involved in the development of substitute decision maker documents [9].

The importance of general practitioners in guiding patients’ ACP is well documented and there are many studies examining how the uptake of ACP in general practice can be promoted [8, 10, 11]. As location of death is unpredictable, general practitioners remain central to ensuring advance care plans and end-of-life care are aligned with patient preferences where medically indicated and appropriate [12]. For people who died in high-income countries between 2010 and 15 [13], 47–60% died within the acute hospital setting [14–16]. In 2017, there was a discrepancy between the proportion of Australians who preferred die at home (70%), and the number who actually did so (50%) [17, 18]. Identifying temporal trends in these data is complicated by both a paucity of population-level data around patient preferences, and the concept that such preferences can change over time [19]. As such, it is important that patients’ preferences are documented and communicated across healthcare settings.

Internationally, various legal rulings, legislations and guidelines outline how ACP is incorporated into medical practice [9, 20–25]. The state law in Victoria, Australia outlines that health practitioners must make a r*easonable effort* to determine the existence of an advance care plan [8]. While the creation of advance care plans is well documented, there is a dearth of literature analysing the communication of documents between parts of the healthcare system [26]. A formal pathway is not often followed, leading to uncertainty as to whether advance care plans that are made in general practice are accessible to hospital staff [27]. In Australia, current methods of ACP document communication from general practice to hospitals vary and include: facsimile, email, post or the patient bringing in their own documents from their place of residence [27].

The importance of ACP document communication has been supported by a High Court ruling in the United Kingdom, determining that all general practitioners must communicate any ACP documents to another treating healthcare service [28]. National registries and health records, such as the National Health Service Digital in the United Kingdom or My Health Record in Australia, may assist in bridging this gap in communication [29, 30] but as at March 2020, the number of ACP documents uploaded to My Health Record was 0.11% (26,920/22,740,000) [31]. Within countries, there are inconsistencies in where and how ACP documents are stored within electronic health records [32–34]. Electronic record system alerts can be used to inform hospital staff that a patient file includes an ACP document [34, 35]. Defining this ‘missing link’ in the ACP process may assist in more people’s preferences being known and respected during end-of-life care.

The aim of this study was to 1) identify the proportion of patients who died in a regional hospital without an ACP document system alert, and 2) determine whether these patients had an uncommunicated ACP document(s) remaining in their corresponding general practice record. The findings of this study will support policymakers seeking to identify strategies to improve the communication of ACP documents across health services and support the representation of patients’ preferences during their end-of-life care.

## Methods

### Study design and participants

A retrospective cohort study of decedents’ hospital and linked general practice records was conducted. This study was based in one large regional public hospital in Victoria, Australia and 35 general practices in the immediate surrounding local government area (LGA). The hospital services a population of over 300,000 people across 58,986 km^2^ [36].

The study sample was patients aged 75 years and over who died in the hospital between 1 January 2016 and 31 December 2017. For the assessment of linked general practice records, only the general practices situated in the same LGA as the hospital were included. The Australian Government Productivity Commission mandates that ACP form part of the general practice Medicare Health Assessment for Older Persons (75 and over), thus forming the rationale for the inclusion of the cohort age group and general practices [37]. This Health Assessment is only applicable to individuals aged over 75 years who lived at home and is a general review of current medical conditions and social circumstances [38]. ACP documents were limited to those legislated in the state of Victoria: advance care plan, Enduring Power of Attorney (Medical Treatment) or Enduring Power of Guardianship [8].

The presence of ACP documents in hospital medical records was assessed via the ACP system alert on the record. The general practice of decedents, who had lived in the immediate surrounding LGA, was noted. Thirty-five local general practice clinics were identified and invited to participate in the study via email and/or phone call to their respective Practice Managers. This invitation was followed by a hand-delivered explanatory statement and consent form. In addition, the lead researcher met with a senior general practitioner from each clinic to explain the study. General practice clinics that had closed or changed owners were excluded as these decedents’ records were no longer accessible. Manual record linkage occurred via the decedent’s name and date of birth, with ACP document data then extracted.

### Data collection tool and extraction

In the absence of a validated tool, a data extraction tool was developed and used to collect information from the general practice records. Key variables of interest were identified from Australian ACP policy documents [8, 18] and peer-reviewed literature [39–41]. These were the type of ACP document; patient gender, age, residential aged care facility (RACF) resident status; length of enrolment at the general practice; and whether they had received a Medicare Health Assessment for Older Persons (75 and over). A RACF in Australia is a purpose-built facility providing accommodation and nursing support [42]. The tool was piloted on 5% of records, resulting in minor modifications to reflect the provisional documents that were found. Data were extracted over a two-week period.

### Data analysis

Following cleaning, the data were tested for normality using the Shapiro-Wilk test [43]. All continuous variables had a non-normal distribution, therefore medians with inter-quartile ranges were presented. Frequencies and corresponding percentages are reported for categorical data. Data were analysed using Statistical Package for Social Sciences for analysis [44].

## Results

**Fig. 1:**
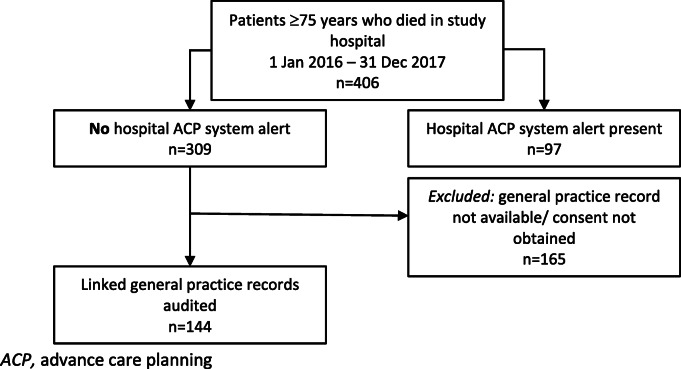
Decedent record analysis. *ACP,* advance care planning

Of the 406 hospital decedents, 309 (76.1%) died in hospital without an ACP document system alert on their hospital record (see Fig. 1).

Of the 35 general practices invited to participate, 17 (48.6%) consented and 15 (42.9%) declined. Two general practice clinics no longer existed, and another had changed ownership which resulted in the current practice not having access to previous patient records. Decedent numbers ranged from 3 to 21 per practice. This led to 144 (46.6%) decedent hospital and general practice records being linked.

The characteristics of general practice decedents are outlined in Table 1. Most decedents were aged 80 years or older. The median time the decedent had attended their nominated general practice clinic was 7.3 years (IQR = 2.0–16.3). One in five of decedents lived in a RACF prior to death in hospital. The remainder were home residents and would have been eligible for a Medicare Health Assessment for Older Persons (75 and over) prior to their death. Of those who were eligible, half had participated in one of these health assessments.

**Table 1:** Characteristics of general practice decedents

*n = 144*		
Gender, n (%)	Female	63 (43.8)
	Male	81 (56.2)
Age (years), median (IQR)		84.9 (81.0–89.4)
RACF resident	Yes	31 (21.5)
	No	103 (71.5)
	Unknown	10 (7.0)
Length of general practice enrolment (years), median (IQR)		7.3 (2.0–16.3)
Medicare Health Assessment for Older Persons (75 and over)^a^	Yes	51 (49.5)
	No	49 (47.6)
	Unknown	3 (2.9)
Time between final *Health Assessment* and death (years)^a^, median (IQR)		1.0 (0.5–1.9)
Time between ACP document creation and death (years)^b^, median (IQR)		2.5 (0.5–4.9)

^a^Only applies to decedents who lived at home prior to hospital admission

^b^Only applies to decedents with an ACP document in their general practice record

Some percentages may not add up to 100% due to rounding

*RACF* Residential aged care facility, *IQR* Interquartile range; *Health Assessment,* Medicare Health Assessment for Older Persons (75 and over); *ACP* Advance care planning

Of the 144 general practice records, 21 (14.6%) contained at least one ACP document that had not been communicated to the study hospital. Of these records, 14 (66.7%) had only one document, either an Enduring Power of Attorney (Medical Treatment) (*n* = 11) or an Enduring Power of Guardianship (*n* = 3). Four (19.0%) records included two ACP documents; two (9.5%) included three documents. Of the four decedents with a written advance care plan, each had an Enduring Power of Attorney (Medical Treatment). Two of these also had an Enduring Power of Guardianship. Most ACP documents were made with the assistance of a lawyer or at their respective general practice. The median time from an ACP document being made to death was 2.5 years (IQR = 0.5–4.9).

## Discussion

To our knowledge, this is the first Australian study to examine the communication of ACP documents from general practice to a hospital. We found most decedents (76.1%) did not have ACP document system alert in their hospital record. Of the 144 corresponding general practice records examined, 14.6% of these records contained an ACP document that was *not* communicated to the hospital nor alerted in their hospital record. It is likely that these documents found only in general practice records were not referenced during the inpatient end-of-life care period.

Previous research has highlighted a discordance between the creation, communication and availability of ACP documents [45]. A 2015 systematic review reported 21–53% of patients had completed an advance care plan but only between 1 and 44% of these were available in emergency department records [26]. Our study only included decedent records; arguably those who we would expect to have the highest rates of ACP completion and availability during end-of-life care. In Australia, there are numerous estimates (14–29%) [39, 46] of the completion of ACP documents in a range of population groups and the prevalence of ACP in hospital patients is 16% [47].

Both patients and practitioners commit considerable time to ACP conversations and creating documents [48]. It is important that these documents are accessible to hospital staff to inform decisions which can enhance the quality of their patient’s end-of-life care. It would be unjust to both the patient and their family if the preferences discussed during an ACP conversation, and any associated documents, are not shared across healthcare settings [49].

Without a sustainable approach to the communication of advance care plans from general practice to hospital, these documents may go unreferenced when clinically required if a patient loses decision making capacity. Although electronic, shared patient records can play a part in facilitating document communication to a hospital service, utilisation and access to these records in general practice and hospitals is not consistent [50]. National electronic health record systems face resistance regarding their overall functionality and the potential for medical information to be accessed by non-authorised third parties [51, 52]. In Australia, an amendment to the *My Health Records Act 2012* allows ACP documents to be uploaded by doctors on behalf of patients [53]. This may alleviate a barrier to uploading documents for older people as this age bracket is known to have poorer computer literacy than their younger counterparts [54]. As noted earlier, in Australia only 0.11% of My Health Record accounts contain an ACP document and not all hospitals can access these records in the acute setting [31]. Though accessibility will improve in time, the issues of communication and the implications of this persist.

In Australia, general practice and hospital records are not electronically linked [55]. The current methods of communicating ACP documents from general practice to hospitals (for example, facsimile, email, postal services and, in some rural areas, manual collection) are not sustainable [27]. Concerns regarding the confidentiality of electronic health records continue and in Australia, facsimile technology is being phased out of healthcare settings [56]. In locations where both the general practice and hospital(s) staff can access electronic health records, such as My Health Record, this mode of communication should be encouraged. Where these are not accessible, other methods need to be utilised. In rural areas, the local hospital is often the only provider of acute healthcare so it may be efficient to extend the use of existing electronic referral software to transmit ACP documents [27]. In metropolitan areas, patients may attend any number of hospitals so My Health Record could improve document availability for patients and doctors.

This study highlights the challenges faced by policymakers charged with integrating paper-based legal documents into an electronic health record system. Internationally, health services are shifting away from paper-based communication [57] and there is evidence that electronic health record systems improve the quality of patient care [58, 59]. However, much of the research on the impact of such records on the cost and efficiency of healthcare has been based within siloed record systems that are limited by a defined set of providers or settings [59]. The greater challenge is how health information is communicated across electronic systems. ACP documents are not only created and stored in different electronic records, but also in legal settings or held by consumers themselves. Such challenges are not exclusive to the Australian setting [57] so this study also provides a foundation for future work in identifying methods and barriers to the communication of ACP documents both within and between healthcare providers.

This study had limitations. It was based in a single regional Australian public hospital and surrounding general practices, so the results may not be generalisable to other healthcare systems or metropolitan settings where patients may attend one of many hospitals. It was beyond the scope of the study to manually check all 409 decedents’ hospital records for documents so there may have been some ACP documents in the hospital records that were not included on the hospital alert system. Due to the low prevalence of ACP documents, we were unable to determine whether an association between a Medicare Health Assessment for Older Persons (75 and over) and ACP documents exists. We only included ACP documents found in general practice records and did not examine RACF records. Although some RACFs have a protocol that a patient’s record be copied when they are transferred to hospital, the presumption that RACFs send ACP documents with the patient may not be actuated [60]. Further research is needed to identify the prevalence of ACP documents remaining in RACF records.

## Conclusions

General practitioners commit time and resources to assist their patients to document their end-of-life care preferences. We found most (76.1%) hospital decedent records did not have an ACP document system alert. Of these, 14.6% of examined general practice records contained an ACP document that was unavailable for use during end-of-life care.

Electronic health record systems may be a long-term solution to the inconsistent communication of ACP documents from general practice to a hospital service. In the short-term, alternative methods of communication are needed to ensure health professionals are aware of documents, and where medically indicated, can respect patients’ preferences during end-of-life care.

## Data Availability

An anonymised form of the dataset used in this study is available from the corresponding author upon request.

## Abbreviations

ACP: Advance care planning
LGA: Local government area
RACF: Residential aged care facility
